# Ultrasound-Guided Hydrodilatation Versus Corticosteroid Injection for Adhesive Capsulitis: A Pilot Randomized Comparative Study

**DOI:** 10.7759/cureus.98413

**Published:** 2025-12-03

**Authors:** Li-Ching Chew, Tyng Yu Chuah, Cassandra Hong, Christopher Wei Yang Liu, Benjamin Sachdev

**Affiliations:** 1 Rheumatology and Immunology, Singapore General Hospital, Singapore, SGP; 2 Duke-NUS Medical School, Duke-NUS Academic Medical Centre, Singapore, SGP; 3 Medicine, Yong Loo Lin School of Medicine, National University of Singapore, Singapore, SGP; 4 Rheumatology, Sengkang General Hospital, Singapore, SGP; 5 Pain Medicine, Singapore General Hospital, Singapore, SGP; 6 Rheumatology, Singapore General Hospital, Singapore, SGP; 7 Rheumatology Unit, Department of Medicine, Hospital Raja Perempuan Zainab II, Kota Bharu, MYS

**Keywords:** adhesive capsulitis, corticosteroid, frozen shoulder, hydrodilatation, intra-articular agents, quickdash score, spadi score, ultrasonography

## Abstract

Objectives

This pilot randomized comparative study aimed to evaluate the feasibility and preliminary effectiveness of ultrasound-guided hydrodilatation (HD) compared with intra-articular corticosteroid injection (IAI) in patients with adhesive capsulitis at six weeks.

Methods

A prospective, single-blind, feasibility randomized comparative study was conducted in a tertiary rheumatology centre. Twenty patients with adhesive capsulitis were recruited and randomized equally to receive HD (n = 10) and IAI (n = 10). The primary outcome measures included the Shoulder Pain and Disability Index (SPADI) score, QuickDASH questionnaire score, pain visual analogue scale (VAS), and range of motion (ROM) at six weeks post-intervention.

Results

Results were reported as mean (± SEM, SD) or median (interquartile range), depending on the distribution of the data. At six weeks, the pain VAS scores were 5 (2, 6) and 1.5 (0.25, 3.75) in the HD and IAI groups, respectively, with P = 0.36. SPADI-Pain scores were 37.8 (17.7-57.8) and 25.3 (5.6-45.0) in the HD and IAI groups, respectively, with P = 0.325. SPADI-Disability scores were 47.3 (26.3-68.3) and 28.5 (8.1-48.9) in the HD and IAI groups, respectively, with P = 0.1602. Median QuickDASH scores were 29.1 (14.5, 41.8) and 13.6 (4.5, 27.3) in the HD and IAI groups, respectively, with P = 0.437. Active and passive ROM also showed no in-between group differences.

Conclusion

No short-term differences between HD and IAI were seen in all the domains studied. Both treatment groups demonstrated improvement in pain and function at six weeks. However, we are unable to conclude that the two treatments have equivalent effectiveness due to the pilot design. The preliminary data may be used to inform larger studies with longer follow-up to determine optimal treatment strategies.

## Introduction

Adhesive capsulitis, also known as frozen shoulder, is a common, functionally debilitating condition characterized by shoulder pain and progressive limitation of both active and passive range of motion (ROM) of the glenohumeral joint [1-3]. It can be divided into primary (idiopathic) or secondary conditions; the latter may be due to either intrinsic (local muscular, tendon pathologies, or rotator cuff tear), extrinsic (abnormalities away from the shoulder, such as cervical radiculopathy or hemiparesis), or systemic conditions (for example, thyroid disorders or diabetes mellitus). The estimated prevalence of adhesive capsulitis is 2-5% of the general population, with a female preponderance occurring between the ages of 40 and 60 years [1,3]. It has been estimated that 20-30% will develop symptoms on the opposing shoulder [1]. Although adhesive capsulitis is considered a self-limiting condition with spontaneous recovery, it typically takes an average of 15 to 36 months to recover. This prolonged course may impact the patient’s quality of life, predominantly due to the functional disability, with chronic symptoms persisting in some patients [3-6]. The pathophysiology of adhesive capsulitis is postulated to be due to a local inflammatory and fibrotic process [3,4,7]. Classification of adhesive capsulitis can be divided into three clinical stages: stage 1 (inflammatory/freezing phase) is characterized by pain and decreased ROM lasting two to nine months, with histology demonstrating hypervascular, hypertrophic synovitis and normal capsular tissue; stage 2 (frozen phase) is characterized by persistent stiffness lasting for four to 12 months and with histology demonstrating hypertrophic, hypervascular synovitis and perivascular and sub-synovial scar formation; stage 3 (thawing phase) is when there is spontaneous recovery or improvement in ROM and pain, and arthroscopy demonstrates fully mature adhesions. This stage typically lasts 12 to 42 months [1,3].

There are no standardized guidelines available for the treatment of adhesive capsulitis, and current treatment goals are to relieve pain and improve shoulder function [3]. Conventional treatment options include measures such as physiotherapy, non-steroidal anti-inflammatory drugs (NSAIDs), intra-articular corticosteroid injections, and hydrodilatation. More invasive treatments such as manipulation under anaesthesia (MUA) and arthroscopic or open capsular release are also recognized treatments [1,3,8]. Despite the myriad of treatment options available, evidence of their efficacy is not well-established. It is unclear which intervention is the most effective.

Conventionally, intra-articular injections with glucocorticoids have been shown in multiple studies to result in the earlier return of pain-free ROM, compared to both placebo and other conservative treatments [9-12]. The alternative treatment method of hydrodilatation has been a topic of interest in recent years, as the former may not be effective in all patients, and its efficacy beyond 12 weeks has not been demonstrated [13]. The systematic review by Koh [13] examined the effectiveness of corticosteroid injections for treating adhesive capsulitis in primary care settings. This review focused on randomized clinical trials to assess the outcomes of corticosteroid injections in managing this condition. Several studies have compared corticosteroid injections to other treatments, such as physical therapy, oral analgesics, or a placebo. Corticosteroid injections were consistently found to provide superior outcomes compared to placebo or no treatment. However, when compared to physical therapy alone, the effectiveness of corticosteroid injection was more variable. Some studies have shown similar outcomes between corticosteroid injections and physical therapy, while others favoured injections for faster relief. Although corticosteroid injections provide effective short-term relief (usually lasting several weeks to a few months), their long-term effectiveness is more limited. Over time, patients who receive corticosteroid injections may be more likely to experience a return of symptoms, suggesting that the benefits of injections diminish as the condition progresses into the more chronic stages (beyond six to 12 months) [13].

Hydrodilatation involves the intra-articular distension of the shoulder joint with a large volume of sterile saline, either with or without the addition of corticosteroids. This process stretches the shoulder capsule, resulting in adhesion breakdown and promoting the remodelling of the capsule. This leads to improved shoulder ROM, especially in those with limited mobility due to the capsular tightening [8]. The local anaesthetic used in the procedure provides immediate pain relief, while the long-term benefit comes from the resolution of the capsular stiffness and inflammation. The improvement provided by the immediate expansion of the joint capsule can be maintained through either sustained hydraulic pressure or physiotherapy. After the procedure, patients are encouraged to undergo physical therapy to maintain and further improve joint mobility [1,3,8]. Furthermore, the combination of hydrodilatation and corticosteroid injections has been shown to reduce the prevalence of long-term deficits [14,15]. Hydrodilatation often helps patients engage more effectively in rehabilitation exercises by improving joint mobility and reducing pain.

Current studies are limited by small sample sizes, lack of blinding, and heterogeneity of study design, rendering it challenging to draw confident conclusions, thus showing a lack of evidence to support one treatment modality over another. This study aims to evaluate the effectiveness of hydrodilatation (HD) compared with intra-articular corticosteroid injection (IAI) in patients with adhesive capsulitis, with pain and function as the primary outcomes. Hence, aiding clinicians in the decision-making process by providing the best available evidence in treating this prevalent and debilitating condition.

## Materials and methods

We conducted a pilot, single-centre, prospective, single-blinded (outcome assessor-blinded), randomized comparative study to evaluate the efficacy of ultrasound-guided shoulder hydrodilatation (HD) compared with conventional intra-articular corticosteroid injection (IAI) for adhesive capsulitis in the short term.

The study was approved by SingHealth Centralised Institutional Review Board (CIRB) (No. 2022/2474) and conducted in accordance with the Declaration of Helsinki. All patients provided written consent before enrolment. Patients were consecutively recruited from the Department of Rheumatology and Immunology and the Pain Management Centre, Singapore General Hospital, between January and October 2023.

Patients were assessed for eligibility according to the following inclusion criteria: (1) adhesive capsulitis or frozen shoulder diagnosed clinically, with or without imaging; and (2) persistent symptoms of pain and restricted ROM for at least three months despite conservative management with physiotherapy and/or analgesia. Exclusion criteria were: (1) previous surgery or shoulder implants of the affected shoulder; (2) history of recent trauma or fracture involving the affected shoulder; (3) age below 21 years; (4) pregnant women; (5) any evidence of ongoing active systemic inflammatory arthritis; (6) allergy to any of the injectates; (7) patients on anticoagulation (warfarin or direct oral anticoagulant); and (8) patients who received any joint injection in the past six months.

The primary outcome measures were assessed at baseline and six weeks post intervention for the following domains: (1) Shoulder Pain and Disability Index (SPADI) score [16], a self-reported questionnaire on pain and disability; (2) QuickDASH score [16], a self-reported questionnaire on upper extremity disability; (3) pain visual analogue scale (VAS) [17,18]; and (4) active and passive ROM [19]. These measurements were performed by two investigators (BS and CTY) who were blinded to the treatments. The measurement instruments utilized in this study are free of charge for non-commercial use and do not require any license. Randomization was performed after the patients had provided written informed consent, and baseline information was collected. Using an online randomization computer software, the research coordinator generated a randomization plan for treatment assignments to the patients. The assignments were then placed into opaque, sealed envelopes secured by the research coordinator. Each patient was allocated to receive either treatment with HD or IAI according to the randomization sequence. As this was an assessor-blinded study, the patients and proceduralists were not blinded, but the assessors were blinded. All procedures were performed under ultrasound guidance.

Techniques for the two different treatments

Hydrodilatation

During the procedure, the patient lay supine on an examination couch, with the affected arm in the neutral position. The proceduralist sat adjacent to the patient, ipsilateral to the shoulder under treatment. All injections were performed under image guidance using a Philips EPIQ ultrasound machine (Philips, Amsterdam, Netherlands) with an L18-12 MHz linear array transducer, consecutively by two rheumatologists (CLC and CH) and one pain medicine anaesthesiologist (CL), all of whom were trained in musculoskeletal ultrasound. An aseptic technique was used for the injections; the skin was sterilized, followed by the application of sterile ultrasound gel on the skin. The needle trajectory did not pass through the sterile gel to prevent the introduction of any gel material into the joint. The transducer was placed at the short axis of the supraspinatus tendon, and the rotator cuff interval and coracohumeral ligament (CHL) were identified. Local anaesthetic (1% lignocaine) was injected subcutaneously into the skin. The therapeutic medication consisted of a mixture of 40 mg triamcinolone (10 mg/ml concentration) and 5 ml of 1% lignocaine, which was diluted with sterile normal saline to a total of 20 ml suspension. There is currently no consensus on what the ideal volume for hydrodilatation is. The standard protocol in our hospital is to use 20 ml, based on our local patients' body size, tolerance, and aiming to achieve distension of the joint capsule. The injection was administered using a 22-G 2-inch needle (Stimuplex A, B. Braun, Melsungen, Germany) introduced below the CHL adjacent to the long head of the biceps tendon, using the in-plane view under ultrasound guidance. The needle trajectory entered from lateral to medial with the doctor positioned adjacent to the patient’s shoulder. The injection was administered very slowly to allow the capsule to accept the given volume. When resistance was met, the injection was briefly halted, and the needle could be repositioned before continuing with the procedure. Rupture of the capsule may occur with this procedure. This is typically felt as a sudden loss of resistance, and visually, on real-time ultrasound, the hypoechoic enlarged capsule may appear to deflate. Some patients may describe a sudden relief of pain or tightness when the joint covering is ruptured. Any adverse reaction that occurred during the procedure was recorded. Following the procedure, the patient was advised to avoid overuse of the treated joint for 24 hours as part of the post-injection care.

Intra-articular Corticosteroid Injection

Intra-articular corticosteroid injection was performed as a control group. The posterior shoulder approach was used. The patient was positioned in the lateral decubitus or semi-prone position with the affected shoulder at the uppermost position, the elbow semi-flexed, and the ipsilateral arm resting on the contralateral shoulder. This position allowed for adequate visualization of the glenohumeral joint. The ultrasound transducer was positioned over the long axis of the myotendinous junction of the infraspinatus tendon to view the contours of the posterior glenoid labrum and posterior portion of the humeral head. The needle trajectory entered from lateral to medial with the doctor positioned adjacent to the shoulder to be injected. The injectate was administered using a 21-G hypodermic needle. Triamcinolone 40 mg (10 mg/ml concentration) mixed with 1 ml of 1% lignocaine was injected into the posterior glenohumeral joint, under ultrasound guidance. Any adverse reaction that occurred during the procedure was recorded. Following the procedure, the patient was advised to avoid overuse of the injected joint for 24 hours as part of the post-injection care.

Statistical analysis

Dichotomous outcome measures are summarized with counts (%). For continuous outcome measures, the Kolmogorov-Smirnov test was performed to determine if the outcomes were normally distributed. For normally distributed data, the results were presented as means and confidence intervals. For statistical testing, the Welch two-sample t-test was used. For skewed data, the results were presented as medians and interquartile ranges (IQR). For statistical testing, the Wilcoxon rank sum test with continuity correction was used. For all statistical tests, a P-value of less than 0.05 was considered statistically significant. An intention-to-treat analysis was performed. All analyses were performed using RStudio 2023.09.1 Build 494 (Posit, Boston, MA).

## Results

Demographics and baseline characteristics of the patients in the HD group and the IAI group are shown in Table 1. No between-group differences were noted, except for duration of symptoms, whereby the IAI group had a longer median symptom duration (12 vs. 7.5 months). The baseline pain scores were similar. None of the participants reported any adverse events during the procedure or follow-up period. Results are reported as mean (± SEM, SD) or median (interquartile range), depending on the distribution of the data.

**Table 1:** Demographic and clinical characteristics of the participants. VAS: visual analogue scale.

	Hydrodilatation (N = 10)	Intra-articular steroid injection (N = 10)
Median age (IQR)	65.5 (57.25, 77)	69.5 (68, 74.8)
Sex		
Female	8 (80%)	6 (60%)
Male	2 (20%)	4 (40%)
Duration of symptoms		
Median (IQR) months	7.5 (3.5, 12)	12 (6, 52.5)
Dominant hand		
Left	1 (10%)	1 (10%)
Right	9 (10%)	9 (10%)
Pain scores		
Baseline VAS scores	5.5 (5, 7.5)	5 (4, 6.25)

Feasibility outcomes

**Figure 1:**
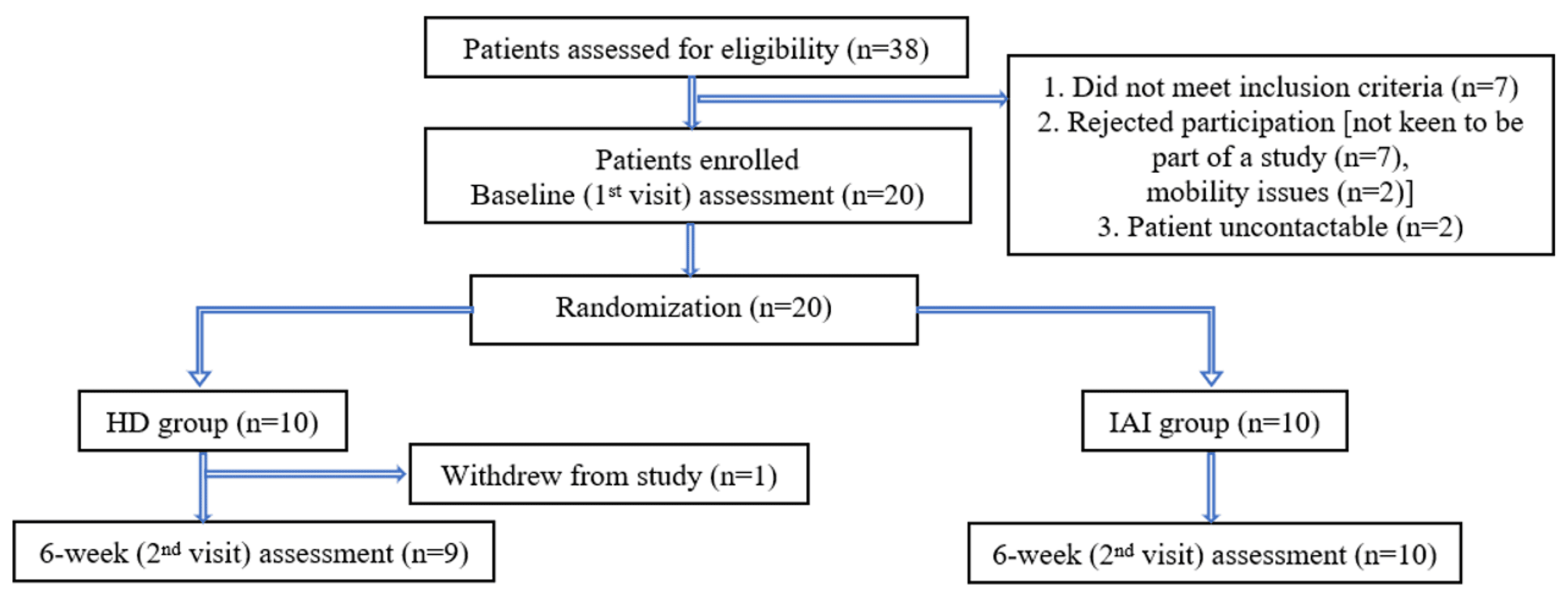
Participant flow chart. HD: hydrodilatation; IAI: intra-articular corticosteroid injection.

A total of 38 patients were screened for eligibility (Figure 1). Of these, seven (18.4%) did not meet the inclusion criteria, nine (23.7%) declined participation (seven were not keen to participate, and two cited mobility issues), and two (5.3%) were uncontactable. Twenty (N = 20) patients were recruited over 10 months. The eligibility rate was 52.6% (20/38 screened participants). All recruited participants were randomized equally into the HD group (N = 10) and the IAI group (N = 10). At the six-week follow-up (second visit), nine participants in the HD group, and all 10 participants in the IAI group completed the assessments. One participant (N =1) in the HD group withdrew from the study after the first visit and was therefore recorded as missing data. The overall retention rate and protocol adherence were high, with 95% (19/20 participants) of the randomized participants completing the scheduled follow-up assessments. The main practical challenges encountered during recruitment and follow-up were the lack of interest in participation and mobility issues faced by the elderly patients, which impacted their ability and convenience to attend the follow-up visits. Finally, an a priori power analysis using G*Power 3.1 (Heinrich Heine University Düsseldorf, Düsseldorf, Germany) for a two-tailed Wilcoxon-Mann-Whitney test (α = 0.05, power = 0.80, effect size d = 0.34, allocation ratio 1:1) indicated that 144 participants per group (total N = 288) were required for future randomized controlled trials.

Pain Visual Analogue Scale (VAS)

A comparative analysis of the pain VAS was conducted among participants in the HD and IAI groups at baseline and six weeks. The baseline VAS pain scores were 5.5 (5, 7.5) and 5 (4, 6.25) in the HD and IAI groups, respectively. At the six-week follow-up, the median pain scores were 5 (2, 6) and 1.5 (0.25, 3.75) in the HD and IAI groups, respectively. However, this difference was not statistically significant (W = 33.5, P = 0.36).

Shoulder Pain and Disability Index (SPADI) Score

We examined the Shoulder Pain and Disability Index (SPADI) scores within the HD and IAI groups at baseline and six weeks. At baseline, the HD group showed a mean SPADI-Pain score of 62.5 (43.4-81.6), while the IAI group demonstrated a mean SPADI-Pain score of 51.4 (33.4-69.3) (t (17.9) = -0.97, P = 0.35). The mean SPADI-Disability score at baseline was 56.7 (40.5-73.0) for the HD group, and 45.1 (27.6-62.7) for the IAI group (t (17.9) = -1.0972, P = 0.29). Additionally, the mean SPADI-Total score at baseline was 80.5 (59.5-101.5) for the HD group, and 67.9 (41.2-94.5) for the IAI group (t (17.1) = -0.84, P = 0.41). At six weeks, the mean SPADI-Pain score for the HD group was 37.8 (17.7-57.8), and the IAI group was 25.3 (5.6-45.0) (t (17.0) =-1.013, P = 0.325). The mean SPADI-Disability score at six weeks was 47.3 (26.3-68.3) in the HD group, and 28.5 (8.1-48.9) in the IAI group (t (16.9) =-1.47, P = 0.1602). Overall, there was a decrease in the SPADI scores at six weeks compared to baseline in both treatment groups. However, no significant difference was observed in the SPADI scores between the IAI and HD groups.

QuickDASH score

QuickDASH questionnaire scores were measured at baseline and at six weeks. At baseline, the median scores for HD and IAI were 41.8 (34.1, 53.6) and 32.7 (11.8, 43.6), respectively (W = 32, P = 0.183). At six weeks, the median scores for HD and IAI were 29.1 (14.5, 41.8) and 13.6 (4.5, 27.3), respectively (W = 35, P = 0.437). Overall, although the QuickDASH scores improved for both groups at six weeks, no significant difference was observed between the two treatment groups.

Range of Motion

Active and passive shoulder range of motion (ROM) were analysed at baseline and six weeks. There was improvement in the ROM for both the HD and IAI groups. However, no significant difference was noted between the two groups (Table 2).

**Table 2:** Group effects of the intervention on active and passive range of motion outcomes. ROM: range of motion.

	Hydrodilatation	Intra-articular steroid injection	P-value
Passive ROM at baseline			
Abduction	82.5 (65, 121.5)	94.5 (85, 127.5)	0.448
Adduction	45 (35.3, 57.5)	45 (32.5, 50)	0.761
Flexion	107.5 (70, 125)	101.5 (66, 123.8)	0.705
Extension	44 (31.3, 65)	50 (46, 57.5)	0.569
Internal rotation	34 (20, 40)	43.5 (31.3, 57.3)	0.517
External rotation	40 (10.3, 60.3)	63.5 (44.5, 85.3)	0.139
Passive ROM at 6 weeks			
Abduction	120 (95, 180)	133.5 (105.8, 173.8)	0.836
Adduction	45 (30, 65)	51 (41.3, 62.5)	0.251
Flexion	120 (112, 160)	140 (128.5, 151.5)	0.652
Extension	55 (30, 60)	52.5 (41.3, 68.3)	0.566
Internal rotation	50 (42, 80)	55 (50, 85)	0.626
External rotation	40 (28, 75)	72 (55, 80)	0.200
Active ROM at baseline			
Abduction	78 (57, 96.3)	86 (72.8, 122.5)	0.472
Adduction	34 (30, 43.8)	36.5 (30, 44.5)	0.909
Flexion	96.5 (70.3, 120)	107.5 (80.5, 128.8)	0.733
Extension	32.5 (30, 45)	51.5 (41, 60)	0.088
Internal rotation	37 (16.3, 65)	40 (25, 53.8)	1
External rotation	28.5 (12.5, 57.5)	62.5 (55.5, 84.3)	0.080
Active ROM at 6 weeks			
Abduction	105 (88, 160)	125 (92.8, 165.5)	1
Adduction	40 (30, 58)	40 (36.3, 56.3)	0.481
Flexion	118 (106, 150)	131.5 (124.3, 147.3)	0.713
Extension	48 (25, 50)	50 (38.5, 60)	0.345
Internal rotation	45 (38, 78)	50 (50, 80)	0.506
External rotation	40 (25, 70)	65 (50, 70)	0.199

## Discussion

There is a myriad of treatment modalities, ranging from conservative approaches, including physiotherapy and analgesic drugs, to corticosteroid injections, hydrodilatation, suprascapular nerve blocks, and surgical interventions such as arthroscopic release and manipulation under anaesthesia; however, none of these have been shown to be consistently effective. The evidence for the efficacy of currently available treatments is not well-established.

The analysis of the results from this study revealed that pain and function, as measured by the pain VAS, SPADI scores, QuickDASH questionnaires, and ROM measurements, achieved numerical improvements in all domains. This suggests that both HD and IAI may be effective in treating adhesive capsulitis. However, no significant difference was noted between the two treatments. To our knowledge, this is the first study in rheumatology patients comparing the effectiveness of HD and IAI, both performed under ultrasound guidance, for the treatment of adhesive capsulitis. Both treatments are often used depending on the severity of the pain and the stage of adhesive capsulitis.

Some studies on the treatment for adhesive capsulitis have supported the use of HD to achieve early improvements in pain and functional recovery. In a randomized controlled trial, distension combined with steroids was compared with steroids alone and concluded that distension with steroids improved ROM at week seven and 12, but pain and functional scores were similar [20]. Another study compared HD with normal saline alone versus steroid injection and found that both groups had similar outcomes immediately post intervention [21]. However, more patients in the HD group experienced sustained improvements in pain and ROM at the four-week assessment. Park et al. showed that although steroid injection with capsular distension had no advantage over steroid injection alone in pain reduction, it achieved better outcomes in ROM, particularly in flexion and internal rotation at four weeks post intervention [22]. Similarly, a prospective randomized study by Yoon et al., which compared outcomes in treatment by intra-articular glenohumeral joint injection, subacromial injection, and HD, showed that the HD group had better outcomes in both ROM and pain relief based on the VAS up to one month, and had improved functional scores up to three months post therapy [23]. All three modalities, however, resulted in similar outcomes when the study period ended at six months. Conversely, other studies have shown no differences in outcomes for pain and ROM for HD versus IAI [24,25]. A recent systematic review and meta-analysis of seven randomized controlled trials showed that although HD demonstrated statistical significance in improving short-term pain relief and ROM, it had a small effect size with a number needed to treat (NNT) of 12; hence, it may not achieve clinical relevance. Furthermore, it did not improve disability levels in the studied population [26].

The comparable outcomes between HD and IAI groups in our study may reflect several factors: (1) shared therapeutic mechanisms (e.g., corticosteroid anti-inflammatory effects in both interventions); (2) insufficient sample size to detect modest differences; and (3) the six-week follow-up was too short to capture potential divergence in treatment effects. A further key consideration was the major difference in the volume of the injectate between the two groups. While both groups received the same corticosteroid dose (40 mg triamcinolone), the larger volume in HD (total 20 ml) produced a mechanical capsular distension, which may disrupt adhesions, stretch the joint capsule, and facilitate improvement in ROM. In contrast, IAI (total 5 ml) relied predominantly on the pharmacologic effect of corticosteroid. The absence of significant between-group differences in the outcomes might suggest that the therapeutic effect was driven by the corticosteroid, or that volumetric distension provided only incremental benefit within the short-term timeframe studied. However, further mechanistic studies examining the relative contribution of the anti-inflammatory effects of corticosteroids versus distension effects are needed. Our findings align with Yoon et al. [23], who reported similar three-month outcomes across the HD, IAI, and subacromial injection groups, suggesting convergence of treatment effects over time. However, unlike Park et al. [22], who observed earlier ROM improvements with HD, our study did not show a similar advantage. This discrepancy may be attributed to the differences in injection techniques, patient characteristics, or chronicity of disease.

Our study has some limitations. Firstly, the sample size was small, as it was a pilot design, to assess the feasibility and determine the sample size for future definitive randomized controlled trials. Based on the effect size observed in this pilot study, a total of 288 patients will be required for the latter in order to be adequately powered. Secondly, as a single-blind study, it was susceptible to performance bias due to the lack of patient blinding. While sham procedures (e.g., superficial skin injections) were considered, they were deemed impractical in this clinical setting. Future confirmatory studies should prioritize double-blinding where feasible, to minimize placebo effects and observer bias. Nevertheless, the assessors who conducted the outcome measures were blinded to the treatments received by the patients. Thirdly, the IAI group had a notably longer median symptom duration (12 vs. 7.5 months). This imbalance could be a potential confounder, as chronicity may affect treatment response and might have biased the results against IAI, assuming longer duration implied a more refractory condition. To mitigate such a confounder in future studies, we recommend: (1) statistical adjustment for baseline differences (e.g., using analysis of covariance (ANCOVA) or regression modelling), and (2) stratified randomization by symptom duration to minimize confounding and ensure comparable group allocation. These measures would enhance the internal validity of comparative efficacy assessments. Furthermore, in some instances, improvement in pain and stiffness associated with adhesive capsulitis may occur gradually over several months to years, as the condition follows a self-limiting natural recovery process. However, there is insufficient evidence to support the complete resolution of this condition without treatment. Fourthly, as the follow-up period was only six weeks, the treatment outcomes and clinical improvement were evaluated in the short term. Extending follow-up to at least three to six months would capture medium-term treatment effects and recovery trajectories. We considered conducting phone consultations with the study patients to inquire about their current pain levels and functional status at three months post intervention; however, the majority of patients did not consent. Finally, while all patients had failed prior physiotherapy before enrolment, the study protocol did not control for post-intervention rehabilitation. The variability in adjunct physiotherapy between participants may have influenced outcomes.

Future studies should standardize post-procedure rehabilitation (e.g., identical physiotherapy regimens for all participants) to isolate the actual treatment effects of HD and IAI. Finally, while functional outcomes, such as the SPADI and QuickDASH, were rigorously assessed, this study did not evaluate patient-reported satisfaction, quality of life (QoL), or willingness to undergo repeated treatment. These metrics are crucial for holistic clinical decision-making, as interventions with similar functional outcomes may differ significantly in patient acceptability. Future studies should incorporate standardized QoL measures (e.g., Short Form-36 (SF-36) and EuroQol 5 Dimension (EQ-5D)) and satisfaction surveys to assess patients’ experiences more effectively.

## Conclusions

In conclusion, while this pilot study found no significant difference between HD and IAI at six weeks post treatment, and both groups demonstrated improvement in pain and function, we were unable to conclude definitively that the two treatments have equivalent effectiveness. The lack of a significant difference observed was likely due to the small sample size of the pilot design. The preliminary data may be used to power larger-scale randomized comparative studies incorporating formal sample size calculations based on the observed effect size, thus informing evidence-based recommendations in the management of adhesive capsulitis. Future research is needed to evaluate the long-term outcomes, optimal dose of injectate, anatomical site of injection, and to identify specific patient subgroups that may benefit preferentially from one treatment over another. Additionally, the inclusion of a placebo (e.g., saline-only group) or a non-intervention control group may aid in differentiating treatment effect from natural disease resolution or placebo response.
